# Rates of Repeated Operation for Isolated Subdural Hematoma Among Older Adults

**DOI:** 10.1001/jamanetworkopen.2018.3737

**Published:** 2018-10-19

**Authors:** Jared Knopman, Thomas W. Link, Babak B. Navi, Santosh B. Murthy, Alexander E. Merkler, Hooman Kamel

**Affiliations:** 1 Department of Neurosurgery, Weill Cornell Medical College, New York, New York; 2 Department of Neurology, Weill Cornell Medical College, New York, New York

## Abstract

**Importance:**

Elderly patients who undergo surgery for an isolated nontraumatic subdural hematoma (SDH) are at major risk for SDH reaccumulation, which can cause further injury and disability. Few population-based studies have examined how often nontraumatic SDH reaccumulates and necessitates repeated operation.

**Objective:**

To determine the rate of repeated operation after evacuation of nontraumatic SDH.

**Design, Setting, and Participants:**

In a cohort study, 2 parallel analyses were performed using data from the National Surgical Quality Improvement Project (NSQIP) from January 1, 2012, through December 31, 2015, and inpatient and outpatient claims data from a 5% nationally representative sample of Medicare beneficiaries from January 1, 2009, to September 30, 2015. In both samples, our cohort comprised patients 65 years or older who underwent surgical evacuation of nontraumatic SDH. Data were analyzed from March 28 to April 13, 2018.

**Main Outcomes and Measures:**

The outcome variable was repeated operation for SDH after the index operation. Survival analysis and Kaplan-Meier statistics were used to calculate cumulative rates.

**Results:**

Among 2 831 274 cases submitted to the NSQIP registry, 515 patients 65 years or older (mean [SD] age, 76.9 [7.4] years; 177 [34.4%] women; 367 [71.3%] white) who underwent craniotomy or craniectomy for nontraumatic SDH were identified. Within 30 days after the index surgery, 37 patients underwent a repeated operation for SDH. The cumulative 30-day mortality rate was 13.8% (95% CI, 11.0%-17.1%), and the repeated operation rate was 7.8% (95% CI, 5.7%-10.7%). Among a cohort of 1 952 305 Medicare beneficiaries, 1534 patients (mean [SD] age, 77.7 [7.0] years; 498 [32.5%] women; 1244 [81.1%] white) who underwent evacuation of nontraumatic SDH were identified. A total of 103 patients underwent a repeated operation within 90 days. The cumulative 30-day mortality rate was 11.6% (95% CI, 10.1%-13.3%), and the repeated operation rate was 4.9% (95% CI, 3.9%-6.2%); the cumulative 90-day mortality rate was 17.6% (95% CI, 15.7%-19.6%), and the repeated operation rate was 7.5% (95% CI, 6.2%-9.0%). The study found no evidence of a significant difference in cumulative repeated operation rates at 90 days between patients whose index surgery was a burr-hole procedure for chronic SDH (11.7%; 95% CI, 6.3%-21.3%) vs a craniotomy or craniectomy (7.2%; 95% CI, 6.0%-8.8%) (*P* = .14 by the log-rank test).

**Conclusions and Relevance:**

In 2 large cohorts of US patients, approximately 5% to 10% of patients who underwent surgery for nontraumatic SDH were required to undergo repeated operation within 30 to 90 days. These results may inform the design of future prospective studies and trials and help practitioners calibrate their index of suspicion to ensure that patients are referred for timely surgical care.

## Key Points

**Question:**

What is the rate of repeated operation for isolated subdural hematoma?

**Findings:**

In this study of 2 cohorts from the National Surgical Quality Improvement Project registry and Medicare beneficiaries, 515 patients older than 65 years and 1534 Medicare beneficiaries underwent surgical evacuation of an isolated subdural hematoma. Of these patients, approximately 5% to 10% required repeated operation within 30 to 90 days.

**Meaning:**

These results highlight the need for prospective studies and clinical trials on better methods to prevent isolated subdural hematoma recurrence and may help practitioners across a variety of disciplines calibrate their index of suspicion to ensure that patients are referred for timely surgical care.

## Teaser

This cohort study uses data from the National Surgical Quality Improvement Project and a nationally representative sample of Medicare beneficiaries to examine how often after surgical evacuation nontraumatic subdural hematoma reaccumulates and necessitates repeated operation among older US adults.

## Introduction

Approximately 125 000 individuals in the United States experience an intracranial subdural hematoma (SDH) each year.^1^ Most cases of SDH occur in those 65 years or older, and SDH in these patients often occurs spontaneously or after minimal trauma and presents in the absence of other types of significant traumatic brain injury, such as cerebral contusions.^1^ Such isolated nontraumatic SDHs are usually surgically evacuated if they are symptomatic or large.^2^ In patients who survive the initial illness and surgery, a major risk is reaccumulation of SDH, which can cause further brain injury and serious long-term sequelae. Unlike patients with SDH attributable to significant trauma, patients with isolated nontraumatic SDH appear to have a lower threshold for subdural bleeding and thus a greater predisposition to SDH reaccumulation,^3^ and reaccumulation is a major challenge in the management of nontraumatic SDH.^4^ Few multicenter prospective studies or clinical trials^5,6^ have been performed to establish treatments for preventing reaccumulation of nontraumatic SDH. Planning for such studies is made difficult by the lack of robust data on the natural history of nontraumatic SDH. Few population-based data are available on how often after surgical evacuation nontraumatic SDH reaccumulates and necessitates repeated operation. We therefore performed a retrospective cohort study using data from 2 large heterogeneous cohorts.

## Methods

### Design

We performed 2 parallel analyses using data from the National Surgical Quality Improvement Project (NSQIP) and a 5% nationally representative sample of Medicare beneficiaries. These 2 sources of data have complementary advantages and offsetting limitations. The NSQIP data provide prospectively collected and clinically detailed information, whereas the Medicare sample allows a more nationally representative analysis, inclusion of burr-hole drainage of chronic SDH and craniotomy procedures, more reliable accounting for out-of-hospital mortality, and a longer follow-up period. We followed the guidelines in the Reporting of Studies Conducted Using Observational Routinely Collected Health Data (RECORD) Statement.^7^ The Weill Cornell Medical College Institutional Review Board approved this study and waived the requirement for informed consent. The NSQIP data were deidentified; the Medicare data are not fully deidentified, but the institutional review board determined that analysis of these data was exempt from review. The NSQIP has developed a national registry to promote quality improvement by providing comparisons of risk-adjusted surgical outcomes at 690 centers.^8^ The NSQIP registry comprises a systematic sample of all patients undergoing surgery under general, spinal, or epidural anesthesia; trauma and transplant cases are excluded. Trained reviewers at centers that participate in NSQIP collect data by medical record review, discussions with health care professionals, and telephone interviews with patients. Data are prospectively abstracted and coded using standardized definitions. All reviewers undergo a uniform training program, and data from each center are subject to audit. The most recent audit showed 98% interrater agreement for all assessed variables,^8^ and this high rate of interrater agreement has been confirmed in independent reviews.^9,10^ For this analysis, we used NSQIP data from January 1, 2012, through September 30, 2015, because these years contained detailed information about repeated operations.

In addition, we used inpatient and outpatient claims data from January 1, 2009, to September 30, 2015, from a nationally representative 5% sample of Medicare beneficiaries. The Centers for Medicare & Medicaid Services provides health insurance to most US residents once they reach 65 years of age. The Centers for Medicare & Medicaid Services makes available to researchers data on claims submitted by practitioners and hospitals during Medicare beneficiaries’ clinical care.^11^ Claims data from hospitals include *International Classification of Diseases, Ninth Revision, Clinical Modification* (*ICD-9-CM*) diagnosis and procedure codes and dates of hospitalization. Physician claims include *ICD-9-CM* codes, *Current Procedural Terminology* (*CPT*) codes, the dates of service, and physician’s specialty. Multiple claims for a given patient can be linked via a unique beneficiary identifier code, thus allowing for a comprehensive and longitudinal analysis of each beneficiary’s care over time. We limited our cohort to beneficiaries with continuous coverage in traditional fee-for-service Medicare (both Parts A and B) for at least 1 year (or until death, if applicable) and no participation in a Medicare Advantage plan. Data were analyzed from March 28 to April 13, 2018.

### Patient Population

In both samples, our cohort comprised patients 65 years or older who underwent surgical evacuation of an isolated nontraumatic SDH. In the NSQIP sample, nontraumatic SDH was based on operative codes that represented craniotomy or craniectomy for SDH evacuation (*CPT* codes 61312 or 61314) and a postoperative diagnosis of nontraumatic SDH (*ICD-9-CM* code 432.1) documented by the neurosurgeon. Patients undergoing burr-hole evacuation as their index procedure were not included in the NSQIP registry.

In the Medicare sample, nontraumatic SDH was defined as *ICD-9-CM* code 432.1 in any hospital discharge diagnosis code position, a definition that has been previously validated to have a sensitivity of 96%, specificity of 89%, and positive predictive value of 94%^1,3^; in a sensitivity analysis, we included only patients with a primary hospital discharge diagnosis of nontraumatic SDH. We defined surgical SDH evacuation based on *ICD-9-CM* procedure codes from the hospital discharge record (*ICD-9-CM* procedure codes 01.09, 01.2, 01.24, 01.25, 01.26, 01.28, 01.31, and 01.51), in keeping with prior studies^1,3^; the date of the first recorded operation was considered the index operative date. Hospital-based *ICD-9-CM* procedure codes for major operations have been previously shown to have excellent sensitivity and specificity.^12^ For subgroup analyses, the index surgery was classified as a craniotomy or craniectomy (*ICD-9-CM* procedure codes 01.2, 01.24, 01.25, 01.26, 01.31, or 01.51) or a burr-hole procedure (*ICD-9-CM* procedure codes 01.09 or 01.28), which is used for evacuation of chronic SDH.

### Measurements

Our outcome variable in both samples was repeated operation for SDH after the index operation. The NSQIP registry prospectively collects data on any repeated operation within 30 days of the index operation. Since 2012, repeated operations have been prospectively classified as being related to the index operation or not, and operative codes are provided that describe the nature of the repeated operation. For this analysis, we defined repeated operation for SDH as a repeated operation that was classified as related to the index SDH evacuation and was accompanied by an operative code for a craniotomy or craniectomy (*CPT* codes 61312 or 61314) or a burr-hole procedure (*CPT* codes 61108, 61154, or 61156). In the Medicare sample, we considered a repeated operation for SDH to have occurred if any of *ICD-9-CM* procedure codes 01.09, 01.2, 01.24, 01.25, 01.26, 01.28, 01.31, or 01.51 were documented after the index surgery date, regardless of whether this repeated operation occurred during the same hospitalization as the index surgery or a different hospitalization afterward.

In the NSQIP sample, we ascertained the demographic covariates of age, sex, and race/ethnicity; the medical comorbidities of diabetes, chronic obstructive pulmonary disease, congestive heart failure, hypertension, renal failure, and tobacco use; the preoperative laboratory values of serum sodium, serum creatinine, white blood cells, platelets, and international normalized ratio; and the date of death, if applicable. The index surgery was also classified as emergency or elective. In the Medicare sample, we used the denominator file to ascertain age, sex, race/ethnicity, and dates of Medicare coverage and death, if applicable. Standard *ICD-9-CM* codes were used to ascertain the Charlson comorbidity index scores,^13^ which were cumulatively carried forward from the start of Medicare coverage until the index SDH surgery.

### Statistical Analysis

We used survival analysis and Kaplan-Meier statistics to calculate cumulative rates of mortality and repeated operation for SDH. Kaplan-Meier cumulative rates were calculated along with 95% CIs. Patients were considered at risk starting on the day of the index surgery and were censored at the time of death, if applicable; in the Medicare sample, patients were also censored if their Medicare coverage ended. In the NSQIP sample, we performed a subgroup analysis stratified by age (older vs younger than the mean age) and whether the index operation was elective or emergency. In the Medicare sample, we performed a subgroup analysis stratified by age (older vs younger than the mean age) and whether the index operation was a craniotomy or craniectomy or a burr-hole procedure. The log-rank test was used to compare cumulative rates between subgroups. We also performed exploratory analyses of other potential risk factors for repeated operation using Cox proportional hazards regression models. The threshold of statistical significance was set at 2-sided α = .05 for all analyses. All analyses were performed using Stata/MP, version 14 (StataCorp).

## Results

**Figure 1.**
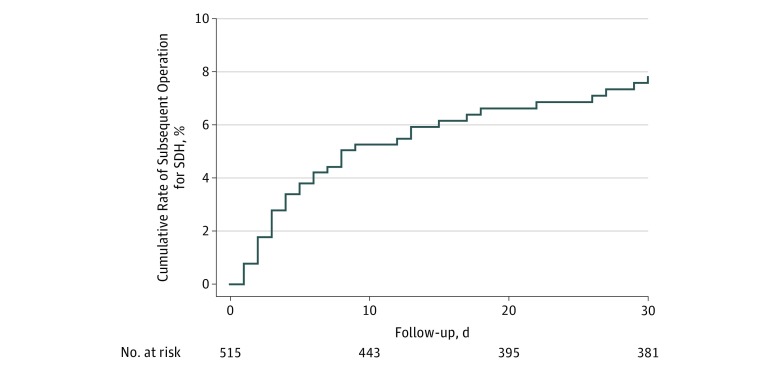
Kaplan-Meier Cumulative Rates of Repeated Operation for Nontraumatic Subdural Hematoma (SDH) in the National Surgical Quality Improvement Program

Among 2 831 274 cases submitted to the NSQIP registry, we identified 515 patients 65 years or older (mean [SD] age, 76.9 [7.4] years; 177 [34.4%] women; 367 [71.3%] white) who underwent a craniotomy or craniectomy for evacuation of a nontraumatic SDH (Table 1). Of these 515 patients, 71 died within 30 days of their index surgery, resulting in a cumulative 30-day mortality of 13.8% (95% CI, 11.0%-17.1%). During the 30 days after the index surgery, 37 patients had a repeated operation for SDH. Accounting for censoring because of death, the cumulative 30-day rate of repeated operation was 7.8% (95% CI, 5.7%-10.7%) (Figure 1). Among those who underwent repeated operation, the median time until repeated operation was 5 days (interquartile range, 2-12 days).

**Table 1.** Baseline Characteristics of Patients With Nontraumatic Subdural Hematoma in the National Surgical Quality Improvement Program Registry, Stratified by Whether Repeated Operation Was Required Within 30 Days^a^

Characteristic	Repeated Operation (n = 37)	No Repeated Operation (n = 478)
Age, mean (SD), y	76.3 (6.7)	76.9 (7.4)
Female	10 (27.0)	167 (34.9)
White race/ethnicity	25 (78.1)	269 (70.4)
Diabetes	8 (21.6)	132 (27.6)
Tobacco use	4 (10.8)	42 (8.8)
Hypertension	29 (78.4)	351 (73.4)
Serum sodium, mean (SD), mEq/L	139 (3)	138 (4)
Serum creatinine, mean (SD), mg/dL	1.0 (0.6)	1.2 (1.1)
White blood cells, mean (SD), /μL	8800 (4400)	9600 (5900)
Hematocrit, mean (SD), %	37.2 (5.9)	36.4 (5.8)
Platelets, mean (SD), ×10^3^/μL	218 (103)	222 (86)
International normalized ratio, mean (SD)	1.1 (0.1)	1.2 (0.4)

SI conversion factors: to convert sodium to millimoles per liter, multiply by 1; creatinine to micromoles per liter, multiply by 88.4; white blood cells to ×10^9^/L, multiply by 0.001; hematocrit to a proportion of 1, multiply by 0.01; and platelets to ×10^9^/L, multiply by 1.

^a^ Data are presented as number (percentage) of patients unless otherwise indicated.

**Figure 2.**
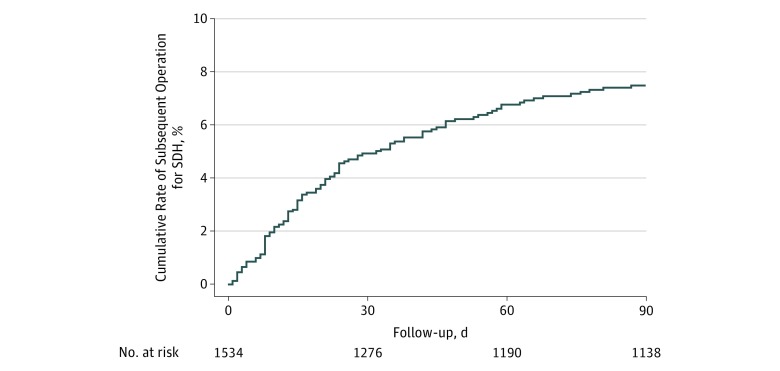
Kaplan-Meier Cumulative Rates of Repeated Operation for Nontraumatic Subdural Hematoma (SDH) Among Medicare Beneficiaries

Among our cohort of 1 952 305 Medicare beneficiaries, we identified 1534 patients (mean [SD] age, 77.7 [7.0] years; 498 [32.5%] women; 1244 [81.1%] white) who had a surgical evacuation of a nontraumatic SDH (Table 2). Of these 1534 patients, 267 died within 90 days after their index surgery, resulting in a cumulative 30-day mortality of 11.6% (95% CI, 10.1%-13.3%) and a cumulative 90-day mortality of 17.6% (95% CI, 15.7%-19.6%). Seventy patients underwent a repeated operation for SDH within 30 days after surgery, and 103 patients underwent a repeated operation for SDH within 90 days after surgery. Accounting for censoring because of death or end of Medicare coverage, the cumulative 30-day rate of repeated operation was 4.9% (95% CI, 3.9%-6.2%), and the cumulative 90-day rate was 7.5% (95% CI, 6.2%-9.0%) (Figure 2 and Table 3). Among those who underwent repeated operation, the median time until repeated operation was 19 days (interquartile range, 8-38 days). Repeated operation rates were similar in a sensitivity analysis that included only patients with a primary hospital discharge diagnosis of nontraumatic SDH.

**Table 2.** Baseline Characteristics of Patients With Nontraumatic Subdural Hematoma in a 5% Nationally Representative Sample of Medicare Beneficiaries, Stratified by Whether Repeated Operation Was Required Within 90 Days

Characteristic	Repeated Operation (n = 103)	No Repeated Operation (n = 1431)
Age, mean (SD), y	78.0 (6.8)	77.7 (7.0)
Female, No. (%)	24 (23.3)	474 (33.1)
White race/ethnicity, No. (%)	88 (85.4)	1156 (80.8)
Charlson comorbidity index score, mean (SD)	2.6 (1.7)	2.8 (1.6)

**Table 3.** Rates of Repeated Operation for Isolated, Nontraumatic Subdural Hematoma in 2 Large Cohorts

Cohort	Cumulative Rates (95% CIs)		*P* Value^a^
	Repeated Operation Within 30 Days	Repeated Operation Within 90 Days	
NSQIP cohort			
All cases	7.8 (5.7-10.7)	NA	NA
Cases stratified by age^b^			
<77 y	8.0 (5.2-12.2)	NA	.80
≥77 y	7.7 (4.9-11.9)	NA	
Cases stratified by urgency			
Emergency index case	8.4 (5.7-12.4)	NA	.59
Elective index case	6.9 (4.1-11.6)	NA	
Medicare cohort			
All cases	4.9 (3.9-6.2)	7.5 (6.2-9.0)	NA
Cases stratified by age^b^			
<77 y	4.7 (3.3-6.6)	7.4 (5.6-9.8)	.89
≥77 y	5.1 (3.8-6.9)	7.6 (5.9-9.7)	
Cases stratified by type of operation			
Burr-hole index case	8.9 (4.4-17.9)	11.7 (6.3-21.3)	.14
Craniotomy index case	4.7 (3.7-6.0)	7.2 (6.0-8.8)	

Abbreviations: NA, not available or applicable; NSQIP, National Surgical Quality Improvement Project.

^a^ *P* values of log-rank tests of the difference in repeated operation rates between the listed subgroups.

^b^ Patients were stratified as older or younger than the mean age, which was approximately 77 years in both cohorts.

We found no evidence of associations for age, operative urgency, or operative type and the rate of repeated operation (Table 3). No significant difference was found in the rate of repeated operation after burr-hole drainage of chronic SDH (11.7%; 95% CI, 6.3%-21.3%) compared with those who underwent craniotomy or craniectomy (7.2%; 95% CI, 6.0%-8.8%) (*P* = .14 by the log-rank test). In exploratory analyses, we found no associations for any of the listed baseline characteristics (Table 1 and Table 2) and time to repeated operation in either cohort.

## Discussion

In 2 large cohorts of US patients, we found that approximately 5% to 10% of patients who underwent surgery for nontraumatic SDH required repeated operation. Most of the risk occurred in the first 30 days, but the risk of repeated operation continued to accumulate during the 3 months after the initial surgery. The risk of repeated operation did not differ substantially after elective vs emergency cases or after craniotomy or craniectomy vs burr-hole procedures. Our findings were robust across several sensitivity analyses.

Prior studies^14,15,16,17,18,19,20,21,22,23,24,25,26,27,28,29,30^ on the natural history of SDH have mostly involved cohorts at single centers, with a few studies^31,32,33^ combining data from 3 centers and 1 study^34^ combining data from 26 neurosurgical training centers. Many of these studies included SDH in the setting of other traumatic brain injuries, which often involve young and otherwise healthy patients, and did not focus on isolated nontraumatic SDH cases in elderly populations, which may have a higher risk of reaccumulation.^3^ Several randomized trials have compared various surgical approaches for draining chronic SDH and their effect on reaccumulation,^5^ but these were small single-center studies, with the largest enrolling 215 patients.^6^ A recent large, multicenter, prospective study^34^ reported outcomes after chronic SDH, but this study involved only select neurosurgical training centers, and most operations were burr-hole procedures. There are few generalizable data from large population-based cohorts regarding repeated operation rates for isolated nontraumatic SDH in elderly patients. In this context, our study provides, to our knowledge, novel and generalizable findings on the rate of repeated operation for nontraumatic SDH in a large heterogeneous sample of elderly patients.

### Limitations

Our findings should be considered in light of several limitations. First, we did not have access to certain clinical data, such as hematoma size, whether the hematoma appeared acute or chronic on imaging, details of the operative approach, and use of antithrombotic therapy before or after surgery. Second, the NSQIP sample may contain multiple episodes that involve the same patient, and this clustering could not be accounted for in our analyses. However, this limitation did not pertain to our Medicare analysis, which had concordant findings. Third, because our outcome was repeated operation, we would not have captured patients who presented with a reaccumulated SDH but were in such poor clinical condition that surgery was judged futile. Thus, the incidence of significant SDH recurrence may be higher than our results indicate. Fourth, for the analysis of Medicare patients, we relied on diagnosis and procedure codes to define our cohort and outcomes, which may result in misclassification. We tried to mitigate this by using previously validated codes, and the findings of that analysis were concordant with our analysis of the NSQIP sample, which involves prospectively collected and audited data.

## Conclusions

Our findings provide a broadly generalizable estimate of SDH repeated operation rates across a variety of US hospitals. We found that a substantial fraction of patients died or required repeated evacuation of nontraumatic SDH. These results may help inform the design of future prospective studies and clinical trials to improve the current state of therapy for nontraumatic SDH. Given the increasing prevalence of SDH with age and the expected aging of the population,^1^ this disease will likely pose an increasing burden during the next several decades. Developing better techniques to prevent SDH recurrence may be a promising target for improving outcomes for this serious disease. Our results may also be helpful to practitioners across a variety of disciplines who care for patients with a history of SDH. Keeping in mind that 5% to 10% of patients with recent surgery for nontraumatic SDH will require repeated operation may help practitioners calibrate their index of suspicion to ensure that patients are appropriately evaluated and referred for timely surgical care.
